# Enhancement of Visuospatial Working Memory by the Differential Outcomes Procedure in Mild Cognitive Impairment and Alzheimer’s Disease

**DOI:** 10.3389/fnagi.2018.00364

**Published:** 2018-11-13

**Authors:** Ana B. Vivas, Antonia Ypsilanti, Aristea I. Ladas, Foteini Kounti, Magda Tsolaki, Angeles F. Estévez

**Affiliations:** ^1^Department of Psychology, CITY College, International Faculty of the University of Sheffield, Thessaloniki, Greece; ^2^Department of Psychology, Sociology and Politics, Sheffield Hallam University, Sheffield, United Kingdom; ^3^Greek Association of Alzheimer’s Disease and Related Disorders, Thessaloniki, Greece; ^4^Department of Neurology, Aristotle University of Thessaloniki, Thessaloniki, Greece; ^5^Departamento de Psicología, Universidad de Almería, Almería, Spain; ^6^CERNEP Research Center, Universidad de Almería, Almería, Spain

**Keywords:** differential outcomes procedure, spatial recognition memory, mild cognitive impairment, Alzheimer’s disease, cognitive intervention

## Abstract

In the present study we investigated the efficacy of the differential outcomes procedure (DOP) to improve visuospatial working memory in patients with Alzheimer’s disease and mild cognitive impairment (MCI). The DOP associates correct responses to the to-be-remember stimulus with unique outcomes. Eleven patients diagnosed with Alzheimer’s disease, 11 participants with MCI, and 17 healthy matched controls performed a spatial delayed memory task under the DOP and a control condition (non-differential outcomes –NOP-). We found that performance (terminal accuracy) was significantly better in the DOP condition relative to the NOP condition in all three groups of participants. AD patients performed worse, and took longer to benefit from the DOP. In line with previous animal and human research, we propose that the DOP activates brain structures and cognitive mechanisms that are less affected by healthy and pathological aging, optimizing in this way the function of the cognitive system.

## Introduction

According to the WHO, 47.5 million of people lived with dementia in 2015 and this number is expected to increase by 59%(75.6 millions) until 2030 (WHO, 2015). Dementia of Alzheimer type (AD) is the most common type as it represents about 60–80% of the cases (OECD/EU, 2016). Worldwide and European initiatives have been put forward in an attempt to ameliorate the negative impact of this age-related neurodegenerative disease, and one of the key actions is promoting better and earlier diagnosis (OECD/EU, 2016). The clinical construct of mild cognitive impairment (MCI) appeared some decades ago, and has been defined as a stage in between healthy aging and early dementia (Petersen, 2016). It is recognized that there are two main types of MCI, amnestic and non-amnestic (Petersen, 2016). That is, individuals who present only memory impairments and those who exhibit other cognitive deficits than memory. Evidence also suggests that amnestic MCI (aMCI) is most likely to progress to dementia of Alzheimer’s type, but the other type might also progress to dementia (Petersen et al., 1999; Arnáiz and Almkvist, 2003).

Since there is currently no accepted pharmacological treatment for MCI (Petersen et al., 2014), and similarly no effective pharmacological treatment for AD (Mangialasche et al., 2010), in the last years there has been an increasing interest in the potential benefits of cognitive training interventions (CCT), which are relatively inexpensive and potentially scalable (see Bamidis et al., 2014 for a review). A recent meta-analysis study (Hill et al., 2017) included 17 (686 participants) and 12 (389 participants) randomized controlled trials on the efficacy of CCT in MCI and dementia patients, respectively. The study reported moderate and significant effect sizes for the efficacy of CCT in improving global cognition, memory, working memory and attention in individuals with MCI. However, there was no evidence in favor of the hypothesis that CCT can benefit individuals already diagnosed with dementia. Also, there were non-significant effects for benefits in executive functions and processing speed in MCI.

A different approach to enhance cognitive performance is to activate processes that are less affected by aging and dementia by applying, for example, basic principles of learning and reinforcement that were discovered early on in animals (e.g., Trapold, 1970; Trapold and Overmier, 1972), instead of training specific cognitive skills that might be already impaired, and thus difficult to recover. Following this rational, Estévez et al. (2001, 2003, 2007) first employed the differential outcomes procedure (DOP) in humans to improve performance in conditional discriminative learning tasks, in which a correct choice response to a specific stimulus-stimulus association is reinforced with a particular outcome. One typical example of an everyday task for senior citizens that requires this type of learning is discriminating prescription pills associated to different health conditions (e.g., yellow pill for hypertension and white pill for cholesterol). During the training with the DOP, the participant would be presented with one of six names of health conditions followed by six different pills (matching-to-sample task). When the adults correctly choose the pill that matches a particular condition, they always receive a specific outcome (e.g., the praise “good job” for hypertension-yellow pill) in the DOP, (see Molina et al., 2015); whereas in the non-differential outcomes procedure (NOP), that is a typical condition of positive reinforcement in this example, there is not a pre-determined and specific link between a particular outcome (the reinforcer) and the correct response to a particular condition-pill association (the stimulus). This apparently very simple manipulation of arranging the outcomes in a task, so that a single and unique outcome is consistently associated with a particular paring of stimuli to be learned, has shown to significantly lessen memory decline in healthy and pathological aging. Thus, in López-Crespo et al.’s (2009) study (see also Savage et al., 1999 for similar results in rats), older adults had better memory accuracy for faces when specific outcomes were used. Actually, memory performance in the group of older adults did not decrease with a longer memory delay (5 vs. 30 s delay) only in the DOP condition. In a later study, Plaza et al. (2012) also demonstrated the benefits of the differential outcomes training in improving memory for faces in a group of 8 patients with dementia of the Alzheimer type. Using the same task employed by López-Crespo et al. (2009), Plaza et al. (2012) reported better performance in the DOP condition only in the group of dementia patients and not in the group of matched healthy controls. That is, the DOP was effective in drastically decreasing impairments of face recognition in dementia patients when short memory delays were employed.

At present, and to our knowledge, the main (and only) theoretical framework proposed to explain the effects observed with the DOP is *the two-memory systems model*, which is based on work conducted by Savage and colleagues in rats (v.g., Savage and Langlais, 1995; Savage and Parsons, 1997; Savage, 2001). According to this model, the unique association, in the DOP, between a particular discriminative stimulus and a specific outcome creates an implicit reward expectancy that is activated with the presentation of the stimulus, the so-called by Savage and colleagues *prospective memory system*. This automatically activated expectancy representation guides and facilitates behavioral choices, and consequently learning and performance. We would like to notice though that the definition of prospective memory in this theory differs from the one used in the cognitive literature with human, where prospective memory refers to future plans and actions which are associated with executive functions. On the other hand, and according to this model, learning under non-differential outcomes conditions depends on maintaining activated the representation of the discriminative stimulus over the delay in the retrospective memory system. At the neural level, prospective and retrospective memory have been associated with distinct neurotransmitters and brain networks (Savage et al., 2004, 2007; Ramirez and Savage, 2007; Savage and Ramos, 2009), which are also differentially affected by healthy and pathological aging (Martorana et al., 2009; Schroeter et al., 2009). Although, we do not know yet the exact processes and brain areas underlying the DOP effects in humans, evidence from animal research with rats strongly suggests that unlike the NOP, the former does not requires the activation of the hippocampus and the cholinergic neurotransmitter system (e.g.,Savage and Parsons, 1997; Savage et al., 2004). Thus, the effectiveness of the DOP in previous studies with healthy older adults as well as dementia patients could be due to this procedure activating processes and brain structures that are less affected by healthy and pathological aging.

To sum up, evidence so far suggests that the DOP can be effective in improving memory for faces in healthy older adults and in patients with AD. In the present study, we further test this hypothesis by investigating the effectiveness of the DOP in improving spatial working memory in a group of individuals with MCI, a group of patients with AD and a group of matched healthy controls. Neuropsychological research on AD has mostly investigated verbal mediated memory (episodic memory and semantic memory), which has long been considered as the most characteristic and earliest cognitive sign of the disease. Comparatively, very little attention has been paid to visuospatial memory, while more recent evidence suggests that spatial memory deficits are present in early stages of AD and may actually constitute early predictors of the disease (see Iachini et al., 2009, for a review). Furthermore, visuospatial abilities play a fundamental role in everyday activities. For instance, being able to find a route and navigate a new environment is essential to maintain independent living. Based on previous studies with the DOP (e.g., Plaza et al., 2012), we expect that spatial working memory will be significantly improved in the group of AD patients when unique outcomes are associated with each target spatial location. Also, since aMCI has a pathology characteristic of early AD (Morris et al., 2001), we expect that spatial working memory will be also improved in this group of participants.

## Materials and Methods

### Participants

Eleven patients with Alzheimer disease (AD group), 11 patients with mild cognitive impairment (MCI group) and 17 healthy controls (HC group) participated in the study. Nine of the MCI participants were diagnosed with multi-domain amnestic MCI (aMCImd) and the remaining two with single domain aMCI. Patients were recruited from the Greek Association of Alzheimer’s Disease and Related Disorders (Alzheimer’s Hellas) in Thessaloniki, and diagnosed by a neurologist. Diagnosis of dementia was made according to the criteria of NINCDS-ADRDA (McKhann et al., 1984). AD patients were categorized in the moderate stage and had a relatively low MMSE score indicating moderate cognitive deterioration (mean MMSE = 14.63). Diagnosis of MCI was made according to the criteria of Petersen (2004) and Winblad et al. (2004), and included neurological and neuroimaging examination, neuropsychological/neuropsychiatric assessment, medical/social history, and blood tests. Scores for the Greek version of Montreal Cognitive Assessment (MoCA; Kounti et al., 2007) are reported in addition to the scores for the Mini-Mental State Examination (MMSE; Folstein et al., 1975) only for MCI patients and control participants (see Table 1), since this instrument was developed to assist in the detection of MCI (Nasreddine et al., 2005). Nonetheless, all the patients completed both screening tools; MoCA was administered after MMSE, with 1 month apart. The group of MCI participants had relatively low MoCA and MMSE mean scores, since several participants were more cognitively impaired although did not classify for the diagnosis of dementia and met Petersen’s criteria for MCI. The term *late* MCI has recently been coined in the literature to refer to a subtype of MCI, which shows a greater cognitive impairment and is more likely to progress to AD (Aisen et al., 2010).

**Table 1:** Demographic variables and mean scores obtained on the Mini-mental State Examination (MMSE) and the Montreal Cognitive Assessment (MOCA) by participants in the study (standard deviations in parenthesis).

	AD group	MCI group	HC group
*n*	11	11	17
Sex (% Female)	60	75	70
Age (years) *p*-value	72.4 (5.30) 0.067	70 (10.9) 0.542	67.5 (7.5)
Years of education *p*-value	7.4 (2.06) 0.314	7.9 (2.4) 0.506	8.5 (2.5)
MMSE *p*-value	14.6 (2.87) **<0.001**	23.6 (0.90) **<0.001**	28.4 (1.42)
MOCA *p*-value		19.1 (0.90) **<0.001**	25.1 (1.43)

*P-values are for comparisons between each group (AD and MCI) and the control group. Comparisons were performed with independent samples Student’s t-tests.*

Cognitively healthy older adults (matched for gender, age, and education) were recruited from Seniors Day Care centres in Neapoli, Thessaloniki. The exclusion criteria for healthy older adults included; (i) any mental health condition that could affect performance on the task (e.g., depression, anxiety, stroke, and insomnia), (ii) intake of psychotropic drugs such as anti-depressants and anxiolytics; and (iii) a score in the MMSE below 24 (Fountoulakis et al., 2000). The study was approved by the University of Sheffield Ethics Committee.

### Stimuli and Materials

The task was designed and run by E-prime v2.0 (Psychology Software Tools, 2012). There were two different versions of the tasks, so that each participant performed the task under differential and non-differential outcomes conditions, with a period of 2 weeks apart. The order of the outcome conditions, and the version-outcome mapping was counterbalanced across participants. The two versions differed only in the geometrical shape that marked target and non-target locations. In one version, the shape consisted of a 2.5 × 2.5 cm white square, whereas in the other version, the shape was a 5 × 2.5 cm lime rectangle. The stimuli were presented on a black background on a touch screen (12.1″ TFT LCD WXGA monitor). The shapes could appear in one of eight positions arranged in a 3 × 3 imaginary rectangle equidistant from the borders. The outcomes consisted of two sets of four pictures of landscapes that were presented at the center of the screen along with the phrase “You may win …” followed by the name of a reinforcer. Each phrase (e.g., “You may win an umbrella”) appeared always with the same picture of landscape. The reinforcers were everyday objects (e.g., umbrella, mug, key ring, a belt, etc.) that were raffled off at the end of the experiment.

### Procedure

Each participant was assessed individually in a quiet room. At the beginning of the first session, each participant was randomly assigned to one of the two outcomes conditions (DOP or NOP). Then, the researcher explained the task orally while a sample trial was shown on the screen, and participants run a practice block of 4 trials. In the DOP condition, each target location was always paired with a specific outcome (e.g., correct responses to the shape appearing on the right upper corner of the screen were always followed by the same landscape picture-phrase). In the NOP condition, a landscape picture-phrase (the outcome) was randomly presented after correct responses to target locations. That is, in this condition each target location was equally often paired with each of the four landscape pictures-phrases. In each version of the task, although the shape could be presented in all eight possible locations across the trials, four locations were never used as targets, and so responses to any of these non-target locations were never reinforced.

**FIGURE 1:**
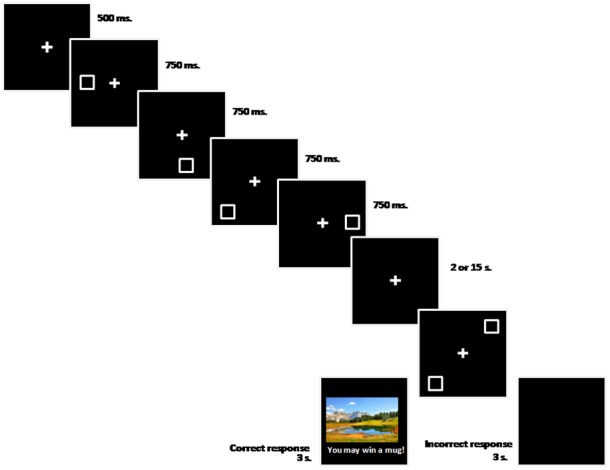
Stimuli sequence (from left to right) used in the experiment.

Each trial sequence (see Figure 1) started with a central fixation point (+) for 500 ms. Then a shape was presented sequentially in four locations (one target and three non-target locations randomly selected for each trial) during 750 ms each time. Right after the last shape disappeared from the screen, a black screen was presented for 2 or 15 s depending on the delay condition. Finally, the probe display appeared on screen until the participant made a respond. The probe display consisted of two shapes (two white squares in one version, and two lime rectangles in the other version of the task): one presented at the target, previously marked, location and the other at a distractor location. In half of the trials the distractor was a non-target location. In the other half, it was one of the three target locations that were not marked during the four-locations sequence. Participants were asked to select the relevant shape (trial-and-error procedure) by touching it on the screen with no time limit. If participants selected correctly the relevant shape, the reinforcer (landscape picture and phrase) was then presented for 3 s. Incorrect responses were followed by a blank screen that lasted also for 3 s. The task consisted of three experimental blocks of 16 trials each.

### Statistical Analysis

Percentages of correct responses for each participant were submitted to a mixed ANOVA with Group (AD, MCI, and HC) as the between-subject factor and Outcomes (DOP and NOP) and Delay (2 and 15 s) as the within-subject factors. Bonferroni *post hoc* test was used for *post hoc* comparisons when appropriate. Latency data did not show any significant effect and therefore only accuracy data are reported. Statistical analyses were performed using SPSS v22.0 and the statistical significance level was set at *p* ≤ 0.05.

## Results

**FIGURE 2:**
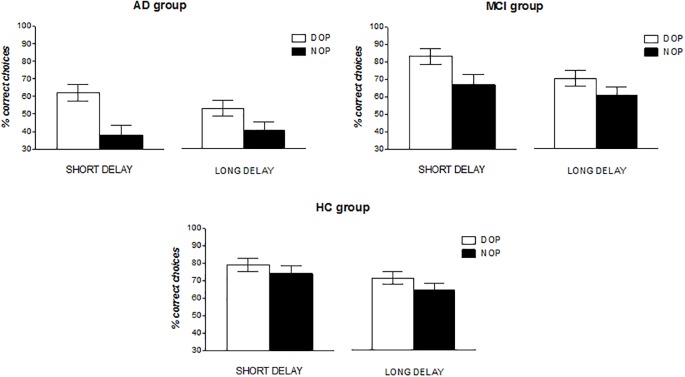
Mean percentage of correct responses as a function of Group (AD, MCI, and HC), Outcomes (DOP and NOP), and Delay (2 and 15 s). Error bars represent the standard error of the mean.

Results from the accuracy data (see Figure 2) analysis showed significant main effects of Delay [*F*(1,36) = 11.56, *p* = 0.002, ${\text{η}}_{\text{p}}^{2}$ = 0.243] and Group [*F*(2,36) = 13.40, *p* < 0.001, ${\text{η}}_{\text{p}}^{2}$ = 0.427]. That is, participants overall were more accurate in the short than in the long delay (67% vs. 61% accuracy for the 2- and 15-s delays, respectively). Bonferroni *post hoc* pair-wise comparisons showed also significant differences between the AD group (49%) and the MCI and HC groups (71% and 73%, respectively; *p*s < 0.001). That is, healthy control and MCI patients were both more accurate than AD patients. Finally, a significant effect of Outcomes was observed [*F*(1,36) = 22.23, *p* < 0.001, ${\text{η}}_{\text{p}}^{2}$ = 0.382] indicating that participants performed the task better in the DOP than in the NOP condition (71% vs. 58% accuracy, respectively). No other effects, nor their interaction, reached statistical significance (*p*s > 0.05).

**FIGURE 3:**
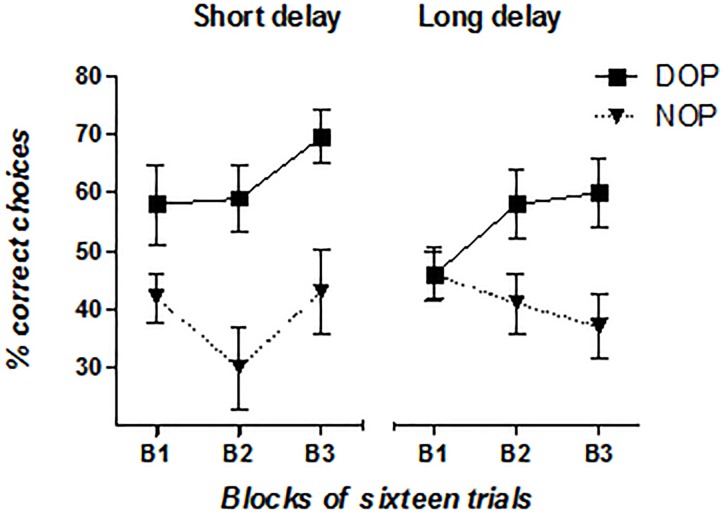
Mean percentage of AD patients’ correct responses as a function of Outcomes (DOP and NOP), Block of trials (B1, B2, and B3), and Delay (2 and 15 s). Error bars represent the standard error of the mean.

Although participants in the three groups appeared to show a better spatial delayed recognition memory in the DOP than in the NOP condition (58% vs. 40%, 77% vs. 65%, and 76% vs. 70% accuracy for the AD, MCI and HC groups in the DOP and NOP conditions, respectively), it is worth noting that overall AD patients’ performance was at chance in the DOP condition (Chi-square = 2.56, *df* = 1; *p* = 0.110). Thus, it appeared that AD patients needed more training with the procedure in order to observe improvements in performance. To test this *ad hoc* hypothesis, we grouped the data from these participants in three blocks of sixteen trials each (see Figure 3) and conducted a repeated measures ANOVA with Outcomes (DOP and NOP), Delay (2 and 15 s) and Block of trials (B1, B2, and B3) as the within-subject factors. Results showed a significant main effect of Outcomes [*F*(1,10) = 15.78, *p* = 0.003, ${\text{η}}_{\text{p}}^{2}$ = 0.612], and a significant Outcomes × Block interaction [*F*(2,20) = 4.56, *p* = 0.023, ${\text{η}}_{\text{p}}^{2}$ = 0.313]. The analysis of the interaction revealed that accuracy linearly increased with blocks of trials only in the DOP condition [*F*(2,20) = 4.16, *p* = 0.031, ${\text{η}}_{\text{p}}^{2}$ = 0.294] (52, 59, and 65% accuracy in blocks 1, 2, and 3, respectively), and that performance was above chance in the last two blocks of trials although this effect was marginal in Block 2 (Chi-square = 3.2, *df* = 1; *p* = 0.072 for Block 2 and Chi-square = 9.0, *df* = 1; *p* = 0.003 for Block 3). However, in the NOP condition performance never reached above chance levels.

## Discussion

In the present study we investigated if the DOP, an easy-to-implement technique, would be effective in improving spatial working memory in people with MCI and AD. Thus, participants in both groups and in a third group of matched healthy controls were asked to remember a cued location after a delay of 2 or 15 s. In the DOP condition, each target location was paired with a unique outcome; whereas in the NOP condition we presented outcomes in a randomized fashion. The results showed that performance was significantly improved under the DOP in all three groups. Still, the group of patients with AD performed significantly worse than the other two groups, which had comparable overall performance. When we conducted further analyses in the group of patients with AD, we found that eventually patients learned how to do the task. That is, they performed significantly above chance in the last block of trials but only under the DOP. These findings replicate and extend the previous finding of a beneficial effect of this procedure in improving memory for faces in healthy older adults (López-Crespo et al., 2009), and patients with AD (Plaza et al., 2012). It is worth noting that the group of AD patients examined in Plaza et al. (2012) differs from the one examined in this present study in terms of severity of cognitive impairments. That is, patients in Plaza et al. (2012) were classified as mild AD; whereas in this study they were categorized as moderate AD. Consequently, the present findings also extend the positive effects of the DOP to AD patients with more advanced cognitive deterioration. This study also shows for the first time that individuals with aMCI (multi- or single domain), which is considered a stage in between healthy aging and dementia, exhibit improved spatial working memory with the DOP. One limitation of the current study was that we did not include and compare other subtypes of MCI such as non-amnestic MCI or the more recent diagnostic differentiation between early and late MCI (Aisen et al., 2010). Thus future studies may investigate other subtypes, since likelihood to progress to AD changes as a function of MCI sub-diagnostic category (Petersen et al., 1999; Arnáiz and Almkvist, 2003; Alexopoulos et al., 2006).

As expected in the control condition (NOP), the performance of AD patients was significantly worse, relative to the group of MCI and HC, and at chance level. This finding is in agreement with evidence suggesting that spatial memory deficits are significant in AD (see Iachini et al., 2009, for a review). However, the performance of the MCI participants did not significantly differ from the HC participants in the control (NOP) condition. This finding does not seem to support the hypothesis that spatial memory deficits may constitute an early marker of AD (Iachini et al., 2009). Given that we employed an experimental task tapping on specific processes, namely delayed visuospatial recognition of locations, future studies should further investigate spatial memory in aMCI.

Although it has been known for decades now that differential outcomes that pair uniquely with a cue-stimulus improve discriminative learning in animals, little is known about the neurocognitive mechanisms behind the DO effect in humans. As discussed earlier on, work conducted with animals suggests that different types of memory processes are activated when learning under differential outcomes as compared to non-differential outcomes (Savage, 2001). That is, it has been found that under NOP conditions the HC is activated, one of the first brain structures affected in AD and MCI (Didic et al., 2013). This brain structure, however, has not been associated to performance under the DOP. Thus, although this study does not offers direct evidence relating this hypothesis, based on the aforementioned studies and the *two memory system* model, we propose that the beneficial effects of the DOP observed in all three groups result from the activation of neural systems and cognitive processes that are less affected by healthy and pathological aging. Future studies should test this hypothesis by additionally investigating how *brain reserve* and neuroplasticity in MCI and AD (Freret et al., 2015), may facilitate the utilization of specific neural networks under differential and non-differential outcomes conditions.

Interestingly, in one of the few studies with humans investigating brain activation in the DOP, Mok et al. (2009) found non-modality specific activation of the posterior parietal cortex (including the posterior cingulate cortex) in the DOP condition, and proposed that this activation could be responsible for the transition from retrospective memory to prospective memory (reward expectancy) processing. Since this area seems to be affected both in AD and MCI, but to a greater extend in AD (Schroeter et al., 2009), this could explain our finding of a later effect of the DOP in the AD patients group.

We would like to conclude highlighting that the present results demonstrate, for the first time, that the way in which the outcomes are associated to the to-be-remember stimulus may greatly affect delayed spatial recognition memory in MCI and AD patients. The inexpensive and easy to implement procedure of applying differential outcomes following correct responses, helped participants to better perform the task. This finding could have significant implications for the everyday life of patients with AD, and older adults with MCI, since spatial skills are crucial to maintain independency (e.g., find the route back home). Since the present study employed a typical experimental-cognitive task, future studies may investigate the effectiveness of the DOP in training spatial skills using more ecological contexts and tasks.

## Author Contributions

AV contributed to the original idea, design of the study, data analysis, and drafted the manuscript. AE contributed to the original idea, design of the study, data analysis, and writing. AY recruited and tested the healthy controls and MCI participants, contributed to data analysis and writing. AL tested Alzheimer’s disease patients and contributed to the writing. FK and MT were responsible for recruitment and testing of AD and MCI patients, for their diagnosis and neuropsychological assessment, and approved the final version to be submitted.

## Conflict of Interest Statement

The authors declare that the research was conducted in the absence of any commercial or financial relationships that could be construed as a potential conflict of interest.
